# Evaluation of the anesthetic depth and bispectral index during propofol sequential target-controlled infusion in dogs

**DOI:** 10.14202/vetworld.2022.537-542

**Published:** 2022-03-08

**Authors:** Matheus Luis Cunha Ubiali, Guilherme Paes Meirelles, Julia Milczewski Vilani, Henrique Erick da Luz, Sabrine Marangoni, Raisa Braul Rodrigues, Ricardo Guilherme D’OCtaviano de Castro Vilani

**Affiliations:** Department of Veterinary Sciences Graduation, Agricultural Sciences Sector, Federal University of Paraná, Curitiba, Brazil

**Keywords:** bispectral index, target-controlled infusion, total intravenous infusion

## Abstract

**Background and Aim:**

The use of anesthetic infusions based on pharmacokinetic values associated with anesthetic plan and bispectral index in dogs have not been well-documented in the literature. This study aimed to evaluate the bispectral index (BIS) change based on pre-propofol and establish clinical anesthetic depth changes during propofol sequential target-controlled infusion (STCI) in dogs with a plasma target of 5 μg/mL.

**Materials and Methods:**

Twenty healthy male dogs aged 1-3 years and weighing 9.8-44 kg were recruited. These dogs were pre-medicated intramuscularly with methadone (0.2 mg/kg) and acepromazine (0.03 mg/kg). After 30 min, propofol anesthetic induction and maintenance were initiated using STCI according to dog pharmacokinetic (PK) parameters. Subsequently, the target plasma concentration of propofol was set at 5 μg/mL for both anesthetic induction and the 120 min maintenance. Then, TivaTrainer v.9.1 software was used to calculate anesthetic infusion rates in a TCI plasmatic concentration mode using the PKs model optimized by covariates for propofol TCI in dogs. The BIS value was recorded every 5 min from the beginning of induction until the end of anesthesia. Finally, analysis of variance was performed on numerical data using the Friedman test, followed by the Bonferroni adjustment (p<0.05).

**Results:**

A statistical difference was observed between the baseline BIS value (T0), with a median value of 84.5 (81-97), and BIS after every 15 min (T15) of inducing anesthesia. Surgical anesthetic depth was also reached in 18 of 20 dogs after 10 min of infusion and in all dogs after 20 min, with a median BIS value of 72 (53-89) at the time of surgical anesthesia depth. Results also showed no BIS variation (p<0.05) between anesthetic moments after anesthetic induction with a substantial amplitude of BIS in the surgical anesthetic depth. Moreover, the maximum depth of anesthesia in all dogs by clinical evaluation was reached after 20 min of anesthesia and then remained stable throughout the anesthetic period.

**Conclusion:**

This study suggested that most dogs (90%) attained a surgical depth of anesthesia within 15 min of STCI onset, with a plasma target of 5 μg/mL and no change in anesthetic depth throughout the period anesthesia lasted. Furthermore, median BIS values remained high even after dogs reached the surgical depth of anesthesia, indicating that the comparison of BIS values of dogs and humans should not be considered for classifying anesthetic and hypnotic depths in dogs.

## Introduction

Propofol is an intravenous anesthetic drug that produces rapid hypnosis, has a short duration of action, and has favorable pharmacokinetic (PK) characteristics for use during continuous infusions. As with most anesthetics, propofol is a gamma-aminobutyric acid (GABA) agonist receptor. In addition, it has a favorable PK and pharmacodynamic (PD) profile, resulting in its widespread use as an intravenous anesthetic [1]. Total intravenous anesthesia (TIVA) involves using injectable drugs intermittently and at constant rate infusions or target-controlled infusion (TCI) to provide balanced analgesia, paralysis, and a state of amnesia or unconsciousness. It can also offer a rapid induction and recovery from anesthesia and decrease post-anesthetic nausea and vomiting [2]. Although TIVA is a recent clinical trend, it is not new. The first documented report of TIVA was in the dog in 1656 [3].

TCI is an anesthetic technique that uses species-specific PK models to calculate rates of anesthetic agent delivered by infusion pumps to achieve and maintain a target plasma concentration. The main difference between TCI and conventional infusions is that TCI slows the infusion rate at regular intervals to account for drug absorption into saturable compartments according to the infusion time and PK model used [4].

The absence of commercial TCI pumps for dogs makes this technique unfeasible in clinical practice. However, some TCI software options accepting user-defined PK parameters and driving selected syringe pumps are available. Examples include Rug Loop I and computer-controlled infusion pump linked to specific models of syringe pumps. In the absence of particular syringe pumps to use the TCI technique in the clinical setting of the veterinary anesthetic routine, this study also proposed infusion pumps with sequential infusion rate functions. However, this absence does not limit the use of the TCI technique exclusively for specific software and pumps, making it possible to be used in clinical routines.

This study aimed to evaluate the possible correlation between variations in bispectral index (BIS) values and anesthetic depth in dogs anesthetized through a more clinically accessible approach to TCI. The hypothesis investigated in this study was the possibility of using sequential TCI (STCI) with a plasma target of propofol (5 μg/mL) to induce and maintain anesthesia in dogs. In addition, we compared the anesthetic plan with the BIS value.

## Materials and Methods

### Ethical approval

The study was approved by the Ethics Committee of the Agricultural Sciences campus of the Universidade Federal do Paraná (Federal University of Paraná, Brazil), in the session of February 28, 2020 (No. 007/2020).

### Study design, period, and location

This prospective experimental study was performed from July 2020 to May 2021. It was conducted in the Veterinary Hospital UFPR, situated in Curitiba, State of Paraná, Brazil.

### Procedures

Twenty male dogs, classified using ASA I (categorization of patients using the American Society of Anesthesiologists patient status scale scoring of 1-5) [5], with their body masses ranging between 9.8 kg and 44 kg, were included in the study. Animals were classified as healthy after clinical and laboratory evaluation, after which they were acclimatized to an individual cage 60 min before starting the anesthetic procedure. Subsequently, all dogs were pre-medicated intramuscularly using a combination of 0.03 mg/kg acepromazine (Acepran 0.2%, Vetnil, SP, Brazil) and 0.2 mg/kg methadone (Mytedom 1%, Cristália, SP, Brazil). Then, anesthesia was induced 30 min later and maintained with propofol (Propotil 1%, BioChimico, RJ, Brazil) using STCI according to the dog PK parameters proposed by Cattai *et al*. [6], using the plasma concentration of propofol (5 μg/mL) as a target. Next, PK data, the volume of distribution, and intercompartmental transfer constants used were obtained from the modeling proposed by Cattai *et al*. [6], considering the following covariates: Weight, age, size, premedication, and sex. PK values were also modeled for each animal and entered the TivaTrainer v.9.1 software (https://www.tivatrainer.com/) using the TCI function plasmatic concentration mode. Afterward, the program generated the individual infusion sequence for each dog’s induction and maintenance of plasma concentration. Later, this sequence was manually entered into the infusion pump (HP TCI, Medcaptain, China) at a sequential flow variation mode before induction. Finally, anesthesia was maintained for 120 min.

### Infusion rates

The first infusion rate of propofol calculated using the software was considerably higher than the others. This result represented a 20 s bolus, raising the blood plasma concentration of propofol from 0 to 5 μg/mL. This rate varied between 1.98 and 2.39 mg/kg/min, representing an initial dose range between 0.66 and 0.8 mg/kg, respectively. After 20 s of infusion, the rate dropped to values between 0.59 and 0.73 mg/kg/mg, representing a reduction of approximately 70% compared with the initial rate. However, after 40 s of infusion, the infusion rate dropped from 1% to 5% every 20 s in the first 8 min. After 8 min from the start of the infusion, the maximum decrease in infusion rate was 3% every 4 min until 40 min of anesthesia. After 40 min from the start of the infusion, the infusion rate remained practically stable from then on (between 0.2 and 0.37 mg/kg/min) for each dog, but with a continuous rate of decrease, less than or equal to 1%, every 4 min until the end of the anesthesia period.

### BIS measurement

The value of BIS was assessed using BIS Vista 3.01 (Aspect Medical Systems Inc., Mansfield, MA02048, USA). First, electrodes (Covidien, Mansfield, MA 02048, USA) were positioned as described by Guerrero and Nunes [7]. Then, to ensure strong brainwave signals, signal quality index and electromyogram values were set at >30. Subsequently, BIS values were recorded immediately before the start of propofol administration (T0) and every 5 min (T5, T10 to T120) until the end of the anesthesia (T120).

### Anesthetics parameters and monitoring

Anesthetic depth was assessed clinically following the modified classification described by Bleijenberg *et al*. [8]. Specifically, superficial anesthesia (palpebral and corneal reflexes present; poor muscle relaxation; and mandibular tone should be present), surgical anesthesia (palpebral reflexes sluggish or absent; jaw muscle tone relaxed; and rostroventral rotation of eyeballs), or deep anesthesia (profound muscle relaxation and eye centrally fixed) were used. After losing the cough reflex, dogs were intubated and mechanically ventilated.

Subsequently, after orotracheal intubation with an appropriately sized tube for each dog, controlled intermittent positive pressure ventilation with 100% inspired oxygen fraction was instituted throughout anesthesia for all dogs. Next, the ventilator (VentPet Plus, RZVET, Brazil) was initially configured with an inspiratory: expiratory (I: E) ratio of 1:2, a respiratory rate (fR) (10 movements/min), and peak pressure (10 cm H_2_O). The fR and peak pressure values were also varied to maintain the end-tidal carbon dioxide pressure between 35 and 45 mm Hg.

In addition, a convection heating system (WarmAir WA7001, Gentherm Medical, USA) was configured to maintain the esophageal temperature between 36°C and 38°C throughout the procedure. Then, heart rate and rhythm were measured by electrocardiogram monitor (TEB-ECGPC VET, Brazil) using a bipolar II lead. After starting anesthesia, direct systolic blood pressures and peripheral tissue oxygen saturation were recorded using a multiparametric monitor (RM1000vet, RZVET). After 60 min of beginning the anesthetic induction, lidocaine (Hypocaína, 20 mg/kg, Hypofarma, MG, Brazil) was injected at the rate of 1.5 μg/kg per application point in intratesticular and pre-scrotal skin incision line. Finally, the surgical procedure (bilateral orchiectomy) started 20 min after executing local blocks. The maximum and minimum times of the surgical procedure were 10 and 20 min, respectively.

### Statistical analysis

Analysis of variance was performed on numerical data using the Friedman test, followed by the Bonferroni adjustment (p<0.05). Then, BIS values were grouped according to time since induction. Furthermore, variations in BIS between anesthetic periods were verified using Friedman’s non-parametric test, followed by *post hoc* with the Wilcoxon test and Bonferroni adjustment at p<0.05. Finally, statistical analyses were generated with the R software Version 4.1.0 (https://www.r-project.org/), using dplyr, rstatix, reshep, PMCMRplus, and ggplot2 packages. Finally, BIS recordings were made 25 times in each animal, totaling 500 measurements.

## Results

**Figure-1:**
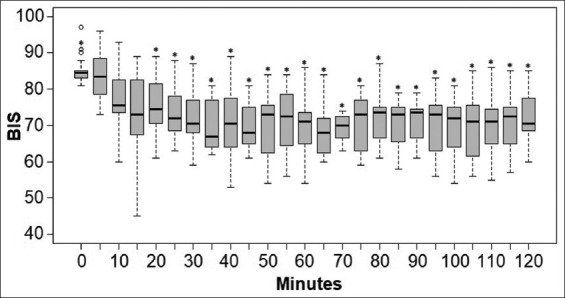
Representation of boxplot of the bispectral index values for the 20 dogs measured at 5 min intervals throughout anesthesia.

A statistically significant difference was observed between the baseline BIS value (T0) and all other measurements made more than 15 min from the start of the infusion. The median baseline BIS value (T0) after the pre-anesthetic medication was 84.5 (81-97). Furthermore, no statistical difference was observed between BIS measurements at T0 and T5, median of 83.5 (73-96, p=1), T10 with a median of 75.5 (60-93, p=0.342), and T15 with a median of 73 (45-89, p=0.07) (Figure-1).

Subsequently, the depth of anesthesia was recorded. At T5, the number of dogs at the superficial and surgical anesthesia depths was, respectively, 11 and 9. Although 2 and 18 were recorded at T10, 1 and 19 were recorded at T15. From T20 till the end of anesthesia, all dogs maintained a surgical anesthetic plane with a median BIS value of 72 (53-89). Nevertheless, no dog was recorded in a deep anesthetic plane at any time.

Figure-1 is a representation of boxplot of the BIS values for the 20 dogs measured at 5 min intervals throughout anesthesia. Statistical differences from T0 by the Friedman test with Bonferroni adjustment (p<0.05) are marked with asterisk (*).

## Discussion

STCI uses TCI simulators in computer programs or a mobile app to calculate a protocol for individual patients. Subsequently, this protocol can be preloaded into an infusion pump in the sequential mode function (or manually administered during anesthesia using a conventional syringe pump). However, new simulations and protocols must be created in real-time when varying the desired target concentrations during anesthesia becomes necessary. If the target plasma concentration is maintained, the anesthetic depth should remain stable throughout the procedure. Nevertheless, anesthetic protocols are designed to maintain hemodynamic stability with an appropriate anesthetic depth in modern anesthesia. Therefore, it can be challenging to measure anesthetic depth [8] since subjective evaluations rely on the anesthetist’s experience. Classification of the anesthetic plane has been described since the 19^th^ century when Arthur Guedel characterized the stages of anesthesia and anesthetic depth according to the degree of hypnosis with ether [9].

Similarly, assessing the anesthetic depth in veterinary practice is achieved by monitoring a combination of physiological variables. Yet, the specific physiological variable evaluated in each case can vary depending on the PK and PD properties of the anesthetic drug used. Hence, muscle relaxation, loss of mandibular tone, and assessment of somatic reflexes in the cranial nerves are also used to assess anesthetic depth. Conversely, it has been reported that cranial nerve reflexes are derived from subcortical and spinal structures and can only provide crude estimations of the degree of consciousness [10]. Moreover, other physiological variables used for monitoring autonomic responses, such as blood pressure and heart and respiratory rates, depend highly on the protocol used.

Therefore, combining the BIS to assist the chosen target in the TCI technique in humans showed reduced mean propofol anesthetic maintenance rates than the non-BIS-assisted TCI technique [11]. Compared with our study, some limitations were observed, the main one being the absence of a control group to link and verify the benefit of BIS associated with the TCI technique in this specific case with STCI. Another correlation between BIS values in dogs using the modified Glasgow Coma Scale score with altered levels of consciousness showed the efficiency of BIS in monitoring consciousness in patients with altered levels of consciousness [12]. However, studies directed to the applicability of BIS in dogs are scarce.

The evaluation of the electrical activity of the cerebral cortex, using measurable and objective variables, has also been used to categorize anesthetic depth and guide the titration of general anesthesia [13]. Besides, BIS measures sub-parameters of brain electrical activity, using human algorithms to analyze wave frequencies during electroencephalographic tracing [14]. This index is measured on a dimensionless unit scale from 0 to 100 [15]. The lower the BIS value, the greater the degree of hypnosis. In humans, although the numerical classification of BIS varies according to the protocol used, it can be used to categorize the anesthetic depth from awake patients through deep sedation and anesthesia to deep hypnosis or isoelectric encephalographic activity [16]. In the study described by Eizadi-Mood *et a*l. [15], the BIS value in humans can be used as a predictor for intubation in unconscious patients, showing a sensitivity of 88% and specificity of 87% for endotracheal intubation, with mean BIS values of 66.47 and 85.21 for patients who needed endotracheal intubation and did not need intubation, respectively. Alternatively, in our study with dogs, median BIS values at the time of intubation were higher. Furthermore, the correlation between results obtained in our study of BIS values and moments of intubation in dogs was not verified due to the high amplitude and higher median BIS values observed in dogs during the intubation. Nevertheless, BIS values, appearing higher in dogs than humans, and categorization according to anesthetic depths have not been established [17].

A difference was observed between BIS values, the above expectations, and results observed in our study compared to reference values in human anesthesia surgical depth [18]. This difference was related to the possible limitations associated with neurophysiologic and anatomic differences between dogs and humans, in addition to the difference in available updates of the proprietary BIS algorithm [19]. However, when comparing BIS values in dogs were demonstrated in the previous studies [8,19], we verified that median BIS values obtained in our study were higher than the previous studies. This difference is proposed to be related to the different anesthetic protocols used. Therefore, the PKs and PDs of drugs and their interactions, including the doses and dosages used in pre-anesthetic medication, anesthetic induction, and maintenance, are proposed to justify this difference.

Burst suppression on electroencephalogram (EEG) is defined as suppression periods longer than 0.5 s during which the amplitude does not exceed 5 mV in humans [20]. The BIS version can contribute to the observed differences in BIS values between the previous studies [8,19] and our study. As observed, high BIS values (>60) were associated with EEG burst suppression patterns [21], suggesting that the algorithm was insufficient to calculate BIS in the presence of isoelectric period or hypothermia [22]. This finding indicated that even though the BIS v.3.01 contained an improved EEG burst suppression detection [23], the algorithm used to calculate the BIS at v.3.01 had some problems during processing of the human EEG in patients with hypothermia, especially at burst and suppression EEG [21]. Among hypnosis monitoring devices, EEG is the most common in detecting electrical activity of the cortex [24]. The last version available for upload in early 2021 was the BIS 3.5 version. Unfortunately, this version is only available on 4-channel BIS monitors.

The reduction in BIS is related to the amount of propofol in the central nervous system (CNS) and hence to the degree of CNS hypnosis. One of the factors determining the quantity of propofol in the CNS is the propofol transfer rate from the blood tissue to the CNS. It depends on several factors, such as the cardiac output, cerebral blood flow, and drug PK properties that determine the transfer rate across the blood-brain barrier (liposolubility and degree of drug ionization, integrity of tissue membranes, and difference in drug concentrations between compartments).

The statistical difference between the observation moments of the BIS value is proposed to be correlated with the chosen target value. For example, higher targets will have higher infusion rates than low targets, consequently increasing the effect of propofol on the CNS and changing the BIS value. Another essential factor correlated with variations in the BIS value is the type of software update used in the BIS monitors. Updates before version 3.01 are susceptible to overestimation of the BIS value in moments of EEG burst suppression detection.

Therefore, the BIS monitor has been made available for more than 20 years for human clinical use and has had an immense impact on academic activity in anesthesiology [25]. However, dog-specific BIS development is required, including a solid and vibrant theoretical basis of the methodology used in forming the sub-parameters used in the EEG algorithms to generate the final BIS value. These considerations would reduce possible failures and errors in using human BIS algorithms for dogs.

## Conclusion

This study suggested that it is possible to use the TCI technique in dogs with anesthetic depth stability throughout anesthesia, even in the absence of specific pumps during the clinical routine. However, the applicability of STCI in the clinical routine should be further studied since some animals are proposed to require more time to reach the surgical depth of anesthesia and thus the use of different plasma targets. Furthermore, BIS values remained constant and in high median values even after dogs attained the surgical depth of anesthesia, demonstrating that comparing BIS values with those in humans should not be considered for classifying anesthetic and hypnotic depths in dogs.

## Authors’ Contributions

RGDC and MLCU: Designed the study. MLCU, JMV, RGDC, GPM, HEL, SM, and RBR: Collected the samples, performed the experiments, and contributed to the original draft. MLCU and RGDC: Review of the manuscript and contributed to the editing of the manuscript. All authors read and approved the final manuscript.
